# Molecular Insights of Fruit Quality Traits in Peaches, *Prunus persica*

**DOI:** 10.3390/plants10102191

**Published:** 2021-10-15

**Authors:** Karpagam Veerappan, Sathishkumar Natarajan, Hoyong Chung, Junhyung Park

**Affiliations:** 3BIGS Co. Ltd., 156, Gwanggyo-ro, Yeongtong-gu, Suwon-si 16506, Korea; karpagam@3bigs.com (K.V.); sathish@3bigs.com (S.N.); hychung@3bigs.com (H.C.)

**Keywords:** *Prunus*, peach, fruit quality, fruit softening, cell wall, transcriptome, sugar, organic acid, texture

## Abstract

Fleshy fruits are the most demanded fruits because of their organoleptic qualities and nutritional values. The genus *Prunus* is a rich source of diversified stone/drupe fruits such as almonds, apricots, plums, sweet cherries, peaches, and nectarines. The fruit-ripening process in *Prunus* involves coordinated biochemical and physiological changes resulting in changes in fruit texture, aroma gain, color change in the pericarp, sugar/organic acid balance, fruit growth, and weight gain. There are different varieties of peaches with unique palatable qualities and gaining knowledge in the genetics behind these quality traits helps in seedling selection for breeding programs. In addition, peaches have shorter post-harvest life due to excessive softening, resulting in fruit quality reduction and market loss. Many studies have been executed to understand the softening process at the molecular level to find the genetic basis. To summarize, this review focused on the molecular aspects of peach fruit quality attributes and their related genetics to understand the underlying mechanisms.

## 1. Introduction

Fruits are matured reproductive organs of an angiosperm that aid in seed protection and dispersal. Essentially, the fruits are made attractive to the vectors through their size, shape, color, odor, taste, and texture. Further, fruits have become an indispensable part of the human diet, and in recent years, fruit consumption has started increasing as there is steady growth of a health-conscious modern society. Eventually, environmental adaptation and domestication resulted in a diverse variety of fruits [1]. Fruits from the Rosaceae family are morphologically distinct like drupe, pome, drupetum, achene, and achenetum. Highly valued fruits such as peaches, nectarines, Japanese plums, apricots, and sweet and sour cherries belong to the genus *Prunus* of the Rosaceae family. Distinctly, peaches *(Prunus persica* (L.) Batsch) and nectarines (*Prunus persica* (L.) Batsch var. nectarina) are the most favorite drupe or stone-type fruits that have high organoleptic properties and nutritional values. The lack of trichome (peach fuzz) in nectarines is its main difference from peaches.

The peach is one of the most economically valued fleshy fruit (after apples and pears) grown in temperate regions of the world. Both the fresh fruit as well as the value-added processed products such as juices, jelly, and/or canned fruits are of consumers’ preferences [2]. The preferences for the fresh intake or the value-added peach products depend on their organoleptic characteristics. The fruit quality attributes such as aroma, sweetness, color, and flesh texture determine the fruit’s organoleptic properties. Thus, there is an inevitable need to study and understand the factors contributing to the fruit quality traits that make peaches more attractive and palatable.

In the process of fruit development, fruit set and fruit growth are important, while fruit ripening is crucial in achieving palate-favorable features. Ripening is genetically regulated, continuously evolving, highly coordinated biochemical and physiological changes occurring in the fruit [3,4]. Mechanistically, in the ripening process, the fleshy fruit turns juicy, aromatic, colorful, and sweet, increasing the edibility. Peaches attain their quality traits during ripening, but in the late stages of ripening, excessive softening occurs, which can have a negative impact on quality attributes, susceptibility to infection, and increased spoilage [5,6,7,8]. Thus, effective post-harvest management of peaches is needed to prevent this senescence-related excessive softening. The exponential growth of next-generation sequencing technologies and bioinformatic analysis made scientists understand and decipher the genetic basis underlying peach ripening for quality improvement and post-harvest management. The small genome size of the peach (265 Mb) makes it a suitable model to study fruit genomics and genetics of fruit ripening in related *Prunus* and Rosaceae fruit crops [9]. The aim of this review is to understand the peach fruit quality attributes and their underlying molecular bases.

## 2. Peach Developmental Stages

The peach is a typical botanical fruit with a single seed surrounded by the lignified hard shell developed from the innermost layer of the ovary wall, followed by the fleshy part developed from the ovary wall [10]. The three layers protecting the seed are the endocarp (stony cover of the seed), the mesocarp (fleshy edible region), and the exocarp (skin). Peach fruit development occurs in four distinctive stages (S1 to S4), with three intense growing stages (S1, S3, S4) and one stage (S2) primarily corresponding to lignification. The mathematical modeling of the fruit growth showed a double sigmoid curve pattern, wherein after the fruit set, stage 1 (S1) is characterized by exponential fruit growth; next, in stage 2 (S2), there is slow growth accompanied by endocarp lignification (stone formation). In stage 3 (S3), the second rapid growth results in increased fruit size, and in stage 4 (S4), the final stage, the fruit reaches full size and ripening starts [11,12]. Other *Prunus* species such as apricots, Japanese plums, and sweet cherries also follow double sigmoid fruit growth patterns [13]. It is a climacteric fruit, thus ripening and softening is ethylene regulated along with the burst of respiration [5,14]. Apart from regulating ripening-related changes in peaches, ethylene signaling also acts as defensive signaling in chilling injury [15].

## 3. Peach Ripening-Related Changes

The peach is a highly nutritional summer fruit with characteristic taste and aroma, grown in temperate areas. It is a climacteric fruit and typically has a short post-harvest phase, undergoing rapid deterioration of nutrients and flavor compositions after ethylene outburst. To prevent the loss of consumer acceptance and market value, understanding the ripening-related sequential biological process such as flavor accumulation, biochemical changes of aroma compounds, and molecular regulation of fruit quality contributors is vital.

### 3.1. Flavor

Consumers’ liking and preference of fruits greatly depend on the flavor, taste, texture, and nutritional values. A wide variety of peach cultivars are developed or under development to meet the need throughout the globe [16,17]. Sweetness and acidity are the two flavor determining factors. Naturally, the sweetness was the commonly preferred consumer demand, while acidity preferences differ around the world. Based on the acidity levels, peaches can be categorized as high-acid and low-acid favored by western and eastern consumers, respectively. Evidence suggests that both acidity cultivars were propagated through natural selection and quality improvement efforts by the breeders [18,19,20,21,22]. The content and composition of soluble sugars and organic acids determine the organoleptic peach flavor attributes. Sucrose, fructose, glucose, and sorbitol are four major sugars contributing to the sweetness, while malic, citric, quinic, tartric, and shikimic acid likely contribute to peach acidity. Malate, citrate, quinate, ascorbate, and oxalate were identified in ripe peach fruit, and malate, citrate, and quinate content account for >95%, becoming major acid contributors [18,23,24].

Synthesis, vacuolar storage, and degradation of organic acids are important processes involved in its accumulation. The differential change in accumulation of organic acids throughout fruit development was observed between high-acid and low-acid cultivars. A dramatic or slight decrease in the total organic acid content was responsible for the final acidity in the ripe fruits [25]. An experimental study deciphering malate, citrate, and quinate content showed varied concentrations through S1 to S4 stages of development. Malate and quinate levels were more predominant than citrate in the S1 and S2 stages in both high- and low-acid cultivars. In the second exponential growth phase, S3, citrate and quinate showed a significant increase. All three organic acids decreased in fully matured and ripened fruit (S4) in all peach fruit, but the degree was higher in low-acid cultivar [6].

The genetics and genomics study on acidity was carried over the years and found that the *D* locus (*D* is for ‘Doux’ meaning ‘sweet’ in French) located at chromosome 5 controls peach acidity and small QTLs for the concentration of sugars. Various GWAS studies narrowed the physical position of the *D* locus but with consistency might be located from 700 to 1000 kbp on Chr5. Though major QTLs for fruit acidity were identified on the *D* locus, few QTLs were also present in chromosomes 1,2, and 6 [22,26,27]. TA (titratable acidity) and pH were the commonly measured acid-related attributes, and low-acid phenotype is the dominant trait in peaches. Though fine mapping and various molecular markers were developed on the *D* locus, a continuous effort was made to identify a possible candidate gene for fruit acidity. Recently, genetic divergence analysis between low-acid eastern cultivars and high-acid western performed sweep analysis and listed potential genes for malate and citrate accumulation. Five putative *ALMT* (aluminum-activated malate transporter 1) were implicated in increased malate content, of which one putative vacuolar malate transporter gene named *PpALMT1* (Pp.LH.06G01819) was transiently overexpressed in peach mesocarp showed increased malate content than vector control mesocarp. *PpALMT1* could be a candidate gene causing divergence in fruit acidity between cultivars [22]. GWAS analysis was performed for TA, pH, malate, and citrate content. One locus (Chr5: 21,714–1,812,811 bp) overlapping with the known *D* locus strongly correlates with phenotyping variance across these four traits. The same study found another strongly associated locus (Chr2: 29,927,641 bp) having a potential contribution to fruit acidity. Zheng et al. [28] proposed five candidate genes, NAD-malate dehydrogenase 1 (*NAD-MDH1*), pyruvate kinase family protein (*PK*), *ALMT9*, pyruvate dehydrogenase kinase (*PDK*), and WRKY DNA binding protein 14 (*WRKY14*) for malate accumulation in peaches. *NAD-MDH1* and *ALMT9* were related to malate synthesis and vacuolar storage, respectively, while *WRKY14* is a transcriptional repressor of *ALMT9*. In high-acid malate predominant cultivars, both *NAD-MDH1* and *ALMT9* were highly expressed, and *WRKY14* was the opposite result. Regarding citrate accumulation, four candidate genes (glutamate decarboxylase-*GAD,* alcohol dehydrogenase-like *1-ADH1, PK,* and *PDK*) were identified to regulate three pathways. Firstly, the *GAD* gene-encoding glutamate decarboxylase catalyzes the decarboxylation of glutamate to gamma aminobutyric acid (GABA), suggestive of citrate degradation. *GAD* gene higher expression in low-acid cultivars coincides with citrate degradation through GABA shunt. Increased pyruvate in the cytosol is transported to mitochondria, wherein citrate synthesis is enhanced. *ADH1* is involved in the ethanol metabolic pathway and catalyzes the reduction in acetaldehyde into ethanol, reducing the concentration of pyruvate in the cytosol. The observed higher expression of the *ADH1* gene in low-acid cultivars explains the mechanism of low pyruvate availability and subsequent low citrate content. In contrast, the *PK* (converts phosphoenolpyruvate (PEP) to pyruvate) gene increases pyruvate in the cytosol, and the higher expression of the *PK* gene was noted in high-acid peaches [28]. The citrate synthesis pathway also plays a role via the *PDK* gene in citrate accumulation. Another candidate gene, *PpRPH* (regulator of pH in peaches), was identified using an integrative omics-based approach [26]. Non-acid genotypes showed a higher level of expression of *PpRPH* than acid type. Transient ectopic expression of the *PpRPH* gene in tobacco leaves and stop-gain SNP in the second exon of *PpRPH* confirms the *PpRPH* candidature in the *D* locus [26].

Sweetness is another important decider of fruit flavor. The content of four soluble sugars, sucrose, fructose, glucose, and sorbitol, determines the organoleptic aspect of peach fruit flavor. Sucrose (~75 to 76%) was found to be a major contributor, followed by fructose (9 to 10%), glucose (8 to 12%), and sorbitol (3 to 4%) [29,30,31,32]. GWAS identified a significant locus associated with sucrose (Chr5: 614,754–1,109,368 bp), fructose (Chr1: 11,738,129–12,006,040 bp), glucose (Chr4: 10,736,973–12,413,438 bp), and sorbitol (Chr6: 22,350,242–22,451,210 bp) [22]. Analysis of improved peach germplasm concluded an increase in the sweetness in improved germplasm than in wild peaches, and it is due to the accumulation of fructose. The candidate gene associated with fructose accumulation was searched, and *PpERDL16* (early response to dehydration 6-like 16) was identified as the causal gene [22]. Comparative genome studies and biochemical analysis on peach flavor components of wild and modern peaches help to understand the molecular bases of new varieties.

### 3.2. Aroma

Aroma perception of food occurs when volatile compounds enter the nasal passage and are perceived by the olfactory system. This retronasal perception of fruit odor is another important consumer acceptance related to fruit quality attributes. Exceptionally, the aroma of peaches is the most favorable, and it is determined by the mixture of volatile organic compounds (VOCs) accumulated during fruit development and ripening. In the Rosaceae family, the peach is the model organism to study volatile compounds. There are about 100 VOCs so far identified in peach fruit, groups such as C6 aldehydes/alcohols (Hexanal, (*E*)-2-hexenal, Benzaldehyde, Benzeneacetaldehyde, Nonanal, 1-Hexanol, and (*E*)-2-hexenol), esters ((*Z*)-3-hexenyl acetate, acetic acid, hexyl ester, benzoic acid, ethyl ester, 2-Hexen-1-ol, acetate, (*Z/E*), benzene, isothiocyanato), lactones (mainly γ- and δ-decalactones, γ-hexalactone, γ-octalactone, δ-dodecalactone), C13 norisoprenoids, ketones (Dihydro-β-ionone, Geranylacetone), and terpenes (linalool, D-Limonene, D-Camphor) are reported to be mainly implicated in the peach aroma. The lactones and esters provide fruity notes, and the C6 compounds contribute to the green sensory notes in the ripening fruit [33,34,35,36,37].

Many natural elements and commercial factors such as hormones, cultivar, harvest time, post-harvest treatment, and storage influence VOC emission. The peach is a climacteric fruit, and thus, the fruit ripening process is ethylene dependent; consequently, inhibition of ethylene biosynthesis or sensitivity in peach fruit affects volatile compound formation [38,39]. The formation of characteristic peach aroma is closely associated with ethylene biosynthesis. Treatment with 1-MCP (1-methylcyclopropenande), an ethylene production inhibitor, negatively affects the aroma of peach [40]. An aroma alteration process was observed during ethylene-dependent ripening, where concentrations of green-note volatile compounds such as n-hexanal, (*E*)-2-hexenal, (*E*)-2-hexenol, and (*Z*)-3-hexenol were decreased. On the other hand, the fruity aroma volatiles, γ-hexalactone, γ-octalactone, γ-decalactone, and δ-decalactone, increased as ripening progressed [40,41]. The typical peach-like aroma is produced by lactones and esters. Similarly, in apricots, γ-decalactone and β-ionone were found to be predominant aromatic compounds contributing to consumer acceptance [42]. Aroma volatiles biosynthesis is derived from the lipoxygenase (LOX) pathway and β-oxidation pathway, both of which are closely related to fatty acid (FA) metabolism. In the LOX pathway, LOX is the first enzyme to catalyze linoleic and linolenic acids to form hydroperoxide precursors, which are further catalyzed by hydroperoxide lyase (HPL) to form C6 aldehydes. Removal of hydrogen from C6 aldehydes was catalyzed by alcohol dehydrogenase (ADH) to produce C6 alcohols. The esters are produced by an esterification reaction catalyzed by alcohol acyltransferase (AAT) between alcohols and acyl-CoAs [43,44,45]. Acyl-CoA oxidase (ACX) is the first and rate-limiting enzyme in the production of lactones originating from the β-oxidation pathway. Various transcriptome analyses to study peach aroma displayed a dynamic change in the expression of genes related to FAs metabolism (*FAD*—fatty acid desaturase), ethylene signal transduction, LOX (*LOX*, *HPL*, *ADH*, *AAT*), and β- oxidation (*ACX*) pathways [40,41,46,47]. Differential expressions of AAT, ADH, PDC, and LOX were observed to have a suspected role in the apricot aroma production [48].

Linalool, an acyclic monoterpene that also imparts sweet, floral, alcoholic note, and terpene synthases (TPSs), is the vital enzyme involved in the formation of linalool [49]. Wei et al. identified that overexpression *PpTPS3* led to the accumulation of linalool while gene silencing led to 66.5% linalool reduction in peach [50]. In addition, transcription factor PpbHLH1 activated *PpTPS3* by directly binding to its promoter region, validating its role in linalool accumulation [50]. Another study showed that UV-B irradiation reduced linalool production through modulation of terpene synthase gene (*PpTPS1*) [51]. Aroma notes depend on the sensing of VOC by the human olfactory system. Perceiving is efficient when VOC is free, while less sensible conjugated forms were also present in peach. Upon ripening or tissue damage, volatiles are liberated from conjugation to free form, then, potentially influencing peach aroma. Majorly, the formation of nonvolatile conjugated molecule occurs through a transfer of sugar molecules to volatile aglycones (glycosylation) catalyzed by UDP-glycosyltransferases (UGTs). UGT enzymes attach a glucose moiety to the hydroxyl position of linalool to form linalyl-β-d-glucoside [51,52]. A search for linalool glycosylation enzymes in peach genome database found *PpUGT85A2* as candidate gene. *PpUGT85A2* glycosylation activity was confirmed using metabolome and transcriptome analyses using different tissue types and fruit developmental stages in various peach cultivars [53]. Thus, bound volatiles such as glycosylated linalool are also the sources of consumers liking peach aroma volatiles.

### 3.3. Fruit Size, Shape, Color

Consumer acceptance at the first glimpse of any fruit is through a visual inspection of its appearance marked by its size, color, and shape. Peaches have a wide range of variations based on the exterior fruit qualities. Based on the shape, peaches and nectarines can either be round or flat shaped. They are yellow- or white-fleshed and red- or pale-skinned based on the presence or absence of color pigments in their flesh and skin. Wild peaches are relatively smaller in size than their modern counterparts. The increase in size might be due to environmental adaptation and, importantly, peach domestication by humans.

Peaches are available in round and flat shapes, where round fruit is the conventional shape, and flat fruits are the mutant of round fruit. Comparatively, flat peach varieties have low titratable acidity, sweet taste, high sugar content, and rich flavor [54]. Flat peaches are called ‘Saturn peach’ or ‘donut peach’ and ‘Pan Tao”, respectively, in western countries and China [55]. Peach fruit shape was reported to be controlled by a single Mendelian factor “S” for “saucer-shaped”. Linage analysis mapped this dominant trait to the distal part of chromosome 6 [56,57,58]. The homozygous dominant mutational genotype “*SS*” makes aborted fruit, whereas ancestral homozygous recessive “*ss*” produces round fruits. Flat fruits carried the heterozygous dominant “*Ss*” genotype [59]. Multiple SNP have been mapped to the “*S*” locus, and candidate genes located at this locus include *PpCAD1* (constitutively activated cell death 1) and *LRR-RLK* (leucine-rich receptor-like kinase) [56,57]. A GWAS study showed that *PpCAD1* is expressed high in round peaches at the fruit maturation stage, and SNP(A-T) within its intron segregates with the flat fruit trait [60]. In contrast, the *LRR-RLK* gene encodes the protein during flower development that ensures the round shape of the ovary and consequently the fruit. Thus, the loss of function of this gene in the heterozygosis allele produces flat fruits [56].

Fruit shape and size are controlled by cell division (cell number), distribution, and expansion (cell size). Guo et al. [55] investigated the cellular basis of flat and round peaches and found that cell numbers in the vertical direction determined shape in early fruit development stages. Specifically, the reduction in cell number in the vertical axis formed a flat shape. Recently, the genome of Rui You Pan 1 (RYP1), a flat peach cultivar popular in China, was reported, and structural variation based GWAS analysis facilitated the identification of a 1.67 Mb heterozygous inversion to cause flat fruit shape [59]. The inversion resulted in the upregulation of *PpOFP2* (ovate family proteins), an adjacent gene located ~3.12 kb upstream of the proximal breakpoint. *PpOFP2* encodes the protein containing the OVATE domain of the OFPS, causing fruit elongation in tomatoes, might also play a causal role in peach shape determination [59,61]. Similarly, resequencing of the genome of the flat peach ‘124 pan’ revealed a ~1.7 Mb chromosomal inversion in the early stages of fruit development, resulting in the flat-shaped trait [62]. Genome analyses predicted *PpOFP1* as a strong candidate gene for the flat fruit trait [63]. An interaction between *PpOFP1* and *PpTRM17* (TONNEAU1 recruiting motifs), an element in an OFP-TRM module, regulates organ shape in plants. Tan et al. [64] studied the genomic differences between the flat peach and its bud sports genome, incorporating an NGS-based approach in addition to GWAS. The result concluded that long loss of heterozygosity (LOH) events occurred at the distal end of scaffold Pp06 of the bud sport genome in the wild-type flat peach used in this study. An associate SNP was found at 26,924,482 bp of scaffold Pp06, where round-shaped fruit have *A/A* genotype while *A/T* or *T/T* genotypes had flat-shaped fruits. Thus, the LOH event results in the loss of the haplotype carrying the T allele and, subsequently, round-shaped fruit were seen in wild-type flat peach cultivars [64].

In peaches, flesh color is one of the attributes implicating the consumer likeliness and nutritional values. Peaches are either yellow or white colored depending on the pigments in the mesocarp, and the ancestral, white-fleshed phenotype is dominant over the yellow one. Peach flesh color is controlled by single locus (*Y*) mapping to linkage group 1 [9,65]. Carotenoid is the major yellow color-producing plant pigment that accumulates in high amounts in yellow fruit chromoplasts. Comparative analysis of yellow-fleshed ‘Redhaven’ peach and its mutant white-fleshed ‘Redhaven Bianca’ suggested that the carotenoid cleavage dioxygenase (*CCD4*) gene was responsible for the carotenoid accumulation in yellow peaches [66]. CCD4 is a catabolic enzyme catalyzing the oxidative cleavage of carotenoids, and reduced expression of it may be responsible for the transition from the white to yellow fruit [67]. Various mutations causing lowered *CCD4* expression have been reported in peaches grown in Japan, Europe, and the United States [68,69,70]. The insertion of a retrotransposon, a simple sequence repeat (SSR), and A → T transversion were the independent mutation events in the *PpCCD4* gene on chromosome 1. Giberti et al. studied the biochemical basis of the CCD4 enzyme reaction and found that other rate-limiting enzymes in the first cleavage reaction of carotenoid might also influence carotenoid accumulation [71].

Anthocyanin is another important plant pigment, and its differential accumulation resulted in distinctive flesh and skin color in peaches [72]. MYB transcription factor (*PpMYB10*) was found to be pivotal for the red coloration of peach skin. A GWAS analysis using structure variation (SV) data deciphered the underlying mechanism of red flesh color around the stone. A 487 bp deletion affecting the *PpMYB10.1* promoter region was found to be correlated with the red flesh around the stone phenotype [63]. Analyses of the deep-colored cultivar ‘Hujingmilu’ (‘HJ’) and a lightly colored cultivar ‘Yulu’ (‘YL’) for diverse anthocyanin accumulation in peel confirmed the responsible role of glutathione S-transferase gene (*PpGST1*) in anthocyanin transport. Consistently, *PpMYB10.1* was required for the transcriptional activation of *PpGST1*; thus, both the genes are indispensable for the red tinge in red peaches [73]. *PavMYB10*, a transcriptional regulator associated with color regulation, was reported as a candidate gene in sweet cherries (*Prunus avium* L.) along with other color QTLs detected on linkage groups (LGs) 4 and 7 [74]. Another key gene-encoding NAC domain transcription factor designated as BLOOD (BL) found in high levels of blood-fleshed peaches activates *PpMYB10.1* by forming a heterodimer with *PpNAC*. Investigation of wild peach *P. mira* (PMHR (blood-colored flesh), PMHY (yellow-colored flesh), and PMHF (milk-white colored flesh)) fruit flesh coloration using integrated transcriptome and metabolome analysis identified 563 differentially regulated genes and 40 differential metabolites including 5 anthocyanin compounds [75]. Peaches grown at different altitudes showed a remarkable change in the skin pigmentation and proteome. High altitude environments doubled the red blushing of the peach skin and concomitant increase in the accumulation of carotenoids and anthocyanins [76]. In summary, the two classes of pigments, carotenoids and anthocyanin, and their regulatory network were responsible for the white, yellow, and red coloration of peach skin and flesh.

Fruit size is quantitively measured in terms of fruit weight, longitudinal and transverse diameters. Evidence from the fossils study confirms there is a steady increase in peach stone sizes from 8000 to 3500 years. The stone size is strongly correlated with fruit size, and human-mediated domestication is the predominant causal factor for increased size [16]. Fruit swelling determines fruit size, and due to its double sigmoidal growth pattern of fruits, two major periods of swelling occur in peach. Both the swelling velocity and duration together determine the final fruit size. The transcriptomic analysis revealed nine most differentially expressed genes were located within the QTLs reported for fruit size. *Ppa004358m* (² -glucosidase) and *Ppa010376m* (expansin-like protein) encode a cell wall protein involved in cell wall alteration in the swelling period, and *Ppa018151m* encodes a pentatricopeptide repeat-containing protein having a profound effect during plant growth and development [60,77]. It is proposed that auxin is the important phytohormone that controls the fruit swelling via cell division and cell cycle checkpoint, eventually cell expansion and proliferation [77]. Yu et al. [21], using selective sweep analysis, identified two cell number regulator (*CNR*) genes (*PpCNR13, PpCNR17*) overlapping with a reported QTL (fruit weight) in the peach genome along with *PpCNR9* and *PpCNR10* contributing to fruit size. The *FW2.2/CNR* family genes were earlier reported to affect fruit size in *Prunus* [78]. Earlier, CNR progeny controlling fruit size and weight were reported in cherries (LG2 and LG6), peaches (LG6), and Japanese plums (LG7) [78,79,80]. GWAS analysis by Cao et al. found that the expression of two expansin genes, *ppa017982m* and *ppa010443m,* positively correlated with fruit diameter, suggesting a control over fruit weight determination. SNP analysis identified an SNP (allelic variant of homozygous C) in the promoter region of *ppa017982m* (chromosome 5 (nucleotide 5,026,380)), presenting higher incidence in small- and medium-size fruits [60].

### 3.4. Fruit Maturity

A comparative analysis of long supply chain peaches (LSCPs), short supply chain peaches (SSCP), and consumer satisfaction have shown that fresh peaches rich in aroma and sugar content were most preferred [81]. Fruit maturity is an important marketing trait, and it is highly variable in peach. Peaches are harvested at physiological maturation (SSCP, matured on trees) and predicted maturation (LSCP, by breeders) to meets the consumer taste demands and as a measure to prevent post-harvest loss, respectively. Gaining more knowledge on maturity time and its related genomics is necessary to meet the market demand. The harvest date, from a commercial perspective, is defined as the day on which peaches attain maturity (decided by growers or by the study) and are harvested. Maturity date (MD) is the duration of time from the first day of the calendar year (Julian day) and harvest date. Peaches usually take two to nine months, between full bloom and harvest date, referred to as the fruit development period (FDP) [81,82]. To prevent excessive post-harvest damage, peaches are usually harvested before physiologically ripe, which is perceived to be less palatable. Peaches can be classified as early (90 days), mid (91–125 days), and late (over 125 days) maturing genotypes based on their FDP [83]. Variations in MD and FDP, such as widening the range of MD/FDP, could benefit the growers to supply fresh fruit for an extended period of time, increase production, find new market avenues, and reduce post-harvest decay. MD and FDP are quantitative traits, and studies have demonstrated two modes of regulation, either by major genes or under the control of QTLs. Major QTLs controlling the MD have been located at LG 4 and 6 of the peach genome [84,85]. QTLs having a major effect on maturity date were also located on LG4 of apricots, Japanese plums, and sweet cherries, showing a greater synteny between the prunus species in this trait [79,84].

Pirona et al. [86] have shown that the QTL in LG4, referred to as *qMD4.1,* behaves as a Mendelian trait for MD in Contender x Ambra (CxA) and WxBy F2 population. Fine mapping and search for candidate genes led to the identification of the *NAC* gene (*ppa008301m*) and a 9 bp INDEL in the last exon, possibly causing early ripening. Elsadr et al. conducted the GWAS, which identified three SNPs on chromosome 4 that were strongly associated with MD/FDP. The SNP covered region (43,067 bp) was termed as MF/FDP locus, and it contains nine genes (*Prupe.4G179100.1*, *Prupe.4G179200.1*, *Prupe.4G179300.1*, *Prupe.4G179400.1*, *Prupe.4G179500.1*, *Prupe.4G179600.1*, *Prupe.4G179700.1*, *Prupe.4G179800.1*, and *Prupe.4G179900.1*) contributing to some aspect of maturity in peaches. The MD/FDP locus was located nearby the qMD4.1 locus [82]. QTL identification studies in relation to bloom date, ripening date/MD, and FDP were performed using a pedigree-based analysis approach in the Texas peach/nectarine germplasm [28]. QTLs on LG1 (172–182 cM), LG4 (48–54 cM), and LG7 (62–70 cM) were verified for BD and a single QTL on the central part of LG4 (40–46 cM) for both RD and FDP [87].

## 4. Fruit Softening, Texture, and Flesh Adhesion

Softening is the last stage in the fruit ripening process where there is a decline in cell wall strength, cell-cell adhesion, accumulation of sugars, organic acids, and VOC. This process is a very crucial agronomic and nutritional trait for marketability and consumer likeliness. In peaches and nectarines, softening continues to occur even when the fruit is detached from the tree. Peaches have a shorter post-harvest period due to their rapid fruit-softening ability. Thus, peach genetics underlying fruit softening were extensively studied to understand the mechanisms to be incorporated in the breeding programs to extend shelf life, preventing economic loss.

Cell wall modification, along with changes in turgor pressure and texture in the mesocarp, is the classic fruit-softening process. The dissolution via depolymerization or solubilization of cell wall matrix polymers, pectin, and glycans occurs by the sequential and coordinated action of a variety of enzymes. Exopolygalacturonase (exoPG), endopolygalacturonase (endo-PG), endo-1,4-β-mannanase, pectin methylesterase (PME), pectin lyase (PL), and β-galactosidase (βGAL) [88] are the most important cell wall-modifying enzymes. In peach, softening is ethylene dependent, which is clearly evident from the 100-fold rise of *PpACO1*(ACC oxidase), an enzyme correlated with the rate of ethylene release [25]. Several studies have been focused on genome-wide identification, gene expression, functional analysis of these enzymes in many fleshy fruits also in peach [89,90].

The fruit cell wall is made up of a network of hemicelluloses, pectin, and structural proteins anchored within the cellulose microfibrils. Fruit softening is the dismantling of cell wall structural polymers, especially pectin, and loss of turgor pressure resulting in a juicy texture. The softening process and the modification of pectin polymers have a positive correlation. Rhamnogalacturonan-I (RG-I), homogalacturonan (HG), and less common rhamnogalacturonan-II (RG-II) are building blocks of pectin polysaccharides. The initializing step in pectin degradation is the removal of a methyl group from HG catalyzed by PME. PG and PEL are the depolymerizing enzyme-cleaving α-1,4 linkages between de-esterified galacturonic acids in HG either randomly or β-elimination, respectively. The side-chain modification is performed by β-Gal, which releases galactose residues in the side chains of RG-I, opening the polymer for further disassembly [91,92].

Texture includes a variety of traits such as firmness, meltiness, and juiciness that directly influence consumer perception of fruit quality. Peaches with wide phenotypic texture variability make an interesting model to study the genetic basis of varied texture phenotypes. Based on the texture, it is classified as melting flesh (MF), non-melting flesh (NMF), and stony hard (SH) trait. Both MF and NMF are characterized by the gradual loss of firmness in early ripening stages, while rapid melting phase and no melting phase are seen in MF and NMF, respectively. SH fruits are very firm and crunchy ever after fully ripe [93].

Peaches are divided as freestone (F) or clingstone (C) based on flesh adhesion to stone (endocarp). However, the degree of adhesion varies, and so some peaches are semi-freestone or semi-clingstone. Combining flesh softening and stone adhesion, peaches can be freestone melting flesh, clingstone melting flesh, and clingstone non-melting flesh [94]. MF is a more dominant trait than NMF, and the major locus (*M*/*m*) located on chromosome 4 harboring two genes from endopolygalacturonase (endo-PG) regulates the MF trait [95]. The stone adhesion is inherited by ‘freestone’ loci (*F*) mapped to the bottom of LG4 [96,97]. Gu et al. [95] showed copy number variants and the presence/absence of two endo-PG genes (*PpendoPGF* and *PpendoPGM*) in the *M* locus as the genetic basis of diversified stone adhesion and melting flesh phenotype. However, the SH trait is independent of the MF/NMF trait, but it presents epistasis. SH texture is due to the null or very low ethylene, and this inability to produce ethylene is due to the lack of indol-3- acetic acid (IAA) content [8]. IAA levels are required for efficient ethylene biosynthesis. Later, studies found that the YUCCA flavin mono-oxygenase gene (*PpYUC11*-like (*ppa008176*)) strongly correlated with the levels of IAA, ethylene biosynthesis, and it is the key gene in SH trait [98]. Further molecular analysis identified an allelic variation in repeat number at a TC microsatellite located in the first intron of the *PpYUC11* gene [8,98]. To find more genetic determinants of the SH trait, an integrated genomic approach involving genome-wide association mapping, transcriptome analysis, and whole-genome resequencing was performed in SH and non-SH accessions, confirming co-segregation of the *PpYUC11*-like intron TC20 allele and the SH phenotype [99]. Tatsuki et al. [100] proposed the indole-3-pyruvic acid pathway (YUCCA pathway) as the main auxin biosynthetic pathway in ‘Akatsuki’ and ‘Manami’ cultivars, and its disruption by insertion of a transposon-like sequence upstream of the *PpYUC11* gene correlates with stony hard phenotype.

An endo-PG gene and protein were differentially expressed in MF and NMF peaches where MF cultivars showed an increased expression than NMF [101]. The differential gene expression is caused by the SNPs at ‘*M* locus’, and mutations in the endo-PG gene result in the decrease/absence of the endo-PG gene in the pericarp and may exhibit NMF texture [101]. Another role of endo-PG was hypothesized, and it states that reduction in firmness and textural changes are two distinct processes related to the cell turgor variation and cell wall disassembly, respectively. The differential pericarp morphology marked with larger apoplastic spaces was observed more in the MF than NMF during softening, and this is coupled with a loss of shape or turgidity. The amount and the number of isoforms of endo-PG are higher in MF pericarp. Endo-PG through pectin solubilization and depolymerization forms large apoplastic spaces filled with solutes, loss of fruit firmness, and loss of cell turgor in MF overall, contributing to the melting flesh [102]. Gabotti et al. studied the role of lignin in the peach flesh texture in NMF (Oro A), MF (Springcrest and‘Sanguinella), and blood-flesh (Sanguinella) cultivars through an enzyme cinnamyl alcohol dehydrogenase (CAD), which catalyzes the reduction in cinnamaldehydes to cinnamyl alcohols serving as a precursor of lignin biosynthesis. Their results illustrate that there might be a role for CAD activity in the determination of NMF phenotype [103]. Expansins are cell wall-loosening proteins shown to be associated with peach softening-related changes. Expansin *PpExp3,* though, has no apparent hydrolytic activity on cell wall polymers, *PpExp3* transcripts accumulated at higher levels in MF cultivar and show ethylene dependency [5].

## 5. Conclusions and Future Perspectives

Greater change in the diet preference toward plant-based products in recent years presents greater demand for fruits and vegetables. Peaches and nectarines are major economical stone fruit praised for their fruit quality, such as taste, aroma, color, texture, shape, and nutritional value. There are several different varieties of peaches bearing a unique blend of fruit qualities that are attained at different ripening stages in a cultivar/genome dependent pattern. Being a climacteric fruit, the fruit ripening is connected with increased respiration rate and ethylene biosynthesis. During fruit ripening, major chemical and physiological changes in texture, sugar/acid ratio, color, and VOC accumulation, occur, increasing palatability. Although peaches achieve most of the consumer needs, the major difficulty is the maintenance of these fruit quality for longer durations. Softening in most of the commercial peaches is rapid, and post-harvest management is challenging. To increase the shelf life, peaches are harvested at commercial maturity, which minimizes the organoleptic quality. Refrigeration is an alternative to prevent or slow down the ripening process, but with peaches, longer storage at low temperatures undergoes chilling injury. To account for the economic loss but retain the fruit qualities, breeders and growers across the world study genetics and physiology of peaches. With the rapid growth of NGS and bioinformatic tools, peach genomes are deeply studied to decipher the genomic influence on the physical trait. Attaining adequate knowledge in peach genetics will help us to understand the molecular mechanism underlying the quality traits. Identifying the molecular markers corresponding to the quality traits will help in parent selection for breeding programs. The resulting progeny can also be marked for heterozygosity or homozygosity for particular or combined traits using genetic markers. In addition, recent increasing interest in fresh consumption will be beneficial for fruits with shorter shelf lives like peaches. Genetic selection of peaches with a wide range of FDP will increase peach supply for longer durations so that an increase in short-chain peaches meets consumer quality preference while retaining the market value.
